# Editorial: Rehabilitation robotics: Challenges in design, control, and real applications

**DOI:** 10.3389/fnbot.2022.957905

**Published:** 2022-07-27

**Authors:** Francisco Romero-Sánchez, Luciano Luporini Menegaldo, Josep M. Font-Llagunes, Massimo Sartori

**Affiliations:** ^1^Departamento de Ingeniería Mecánica, Energética y de los Materiales, Escuela de Ingenierías Industriales, Universidad de Extremadura, Badajoz, Spain; ^2^Programa de Engenharia Biomédica, COPPE, Universidade Federal do Rio de Janeiro, Rio de Janeiro, Brazil; ^3^Biomechanical Engineering Lab, Department of Mechanical Engineering, Research Centre for Biomedical Engineering, Universitat Politècnica de Catalunya, Barcelona, Spain; ^4^Health Technologies and Innovation, Institut de Recerca Sant Joan de Déu, Esplugues de Llobregat, Spain; ^5^Department of Biomechanical Engineering, University of Twente, Enschede, Netherlands

**Keywords:** wearable robots, exoskeletons, exosuits, rehabilitation, human augmentation, neuromusculoskeletal modeling, assistive devices

## Introduction

In the last decade, research focused on rehabilitation robotics has progressed from proposing restricted or rigid solutions in a clinical setting to portable devices compliant with the user and also adapted to the their requirements, based on their disability and the rehabilitation training program. Novel techniques have inspired the evolution of rehabilitation devices from hard and bulky to soft, lightweight, and fully wearable. For example, biologically inspired actuators have relaxed the constraint of having to rely on rigid supports, as the skeletal system can be used to that end. Furthermore, the use of synergies havehas led to a reduction of in the number of actuators and improved their control. Moreover, the latest advances in modeling and simulation have allowed for assessing and compensating for fatigue, as well as simulating the use of assistive devices out of a clinical environment. All these research achievements have enabled a new generation of portable rehabilitation devices. In the present Frontiers Research Topic, novel techniques for the design, simulation, sensing, and control of rehabilitation devices are presented for rehabilitation devices such as powered exoskeletons, neuroprostheses, and equipment for moving the rehabilitation environment out of the clinical setting.

## Challenges in design, control, and real applications

A key challenge in rehabilitation robotics is to create devices that facilitate positive neuroplasticity in the intact moving human *in vivo*. That is, robots that provide as-least-as-needed assistance to neuromuscular targets while promoting voluntary execution of functional movements. In this context, the robot must be transparent and intuitive to prevent an increase of cognitive loads during the rehabilitation process. This objective is not easy to accomplish, as there are many challenges to overcome and many approaches to tackle. Examples of that, among others, are include the simulation of the human-machine interaction, the design and control of the rehabilitation device, the validation in a clinical environment, or the adaptation of the neuromusculoskeletal system to the rehabilitation process. Based on them, we present the contributions to this Research Topic according to the aforementioned challenges, although they are usually found intertwined in this research field.

### Simulation

Simulation-based studies in rehabilitation robotics provide a time- and cost-effective approach to preview real world scenarios. In this sense, Febrer-Nafría et al. simulate crutch-orthosis-assisted walking to choose the optimal active orthosis controller parameters for a specific subject. Their findings improve the traditional trial-and-error approach to select the best maximum knee flexion angle. The simulation provides optimal values to achieve a more balanced assisted gait pattern, making the process fully personalized to the patients' needs and less time-consuming. Cuerva et al. show a predictive simulation study to improve control in power-assisted wheelchairs. Their work investigates the advantages and disadvantages of an impedance control strategy, which that is more natural and effective than other alternatives. They employed predictive simulations of locomotion with power-assisted wheelchairs in different scenarios by using a realistic physiological model of the user's musculoskeletal system and its interaction with the wheelchair. Their results confirm this control strategy as the most useful, but their simulations also found a waste of energy during the propulsion cycle.

### Design

The personalisedpersonalized design of rehabilitation devices has been a challenge for researchers worldwide. Design requirements are different depending on the body segment part to be treated. In addition, these design specifications may change depending on the neuromuscular impairment. For example, assisting a patient with spinal cord injury (SCI) is not the same as helping a patient who has suffered a stroke. In addition, the designed device must be focused on the user's needs. It also must consider the evolution of the rehabilitation process, which may imply adaptations in the structure of the device or its control. In this Research Topic, Laffranchi et al. present the TWIN, a modular exoskeleton for SCI subjects. In their work, users' needs drove an iterative process to improve the system's design and construction. Sensing and control approaches are also presented. Supervised tests in a clinical setting demonstrated a stable gait pattern for rehabilitation, improving cost effectiveness. Regarding the upper limbs, Secciani et al. propose an original mechatronic design of a hand exoskeleton for both home assistance and telerehabilitation. It uses a real-time intention detection algorithm, but can also perform exercises preset by therapists in remotely supervised or unsupervised rehabilitation sessions. Surface electromyography (sEMG) signals are used to detect the user's intention, leading to a customizable, compliant, and comfortable design. In the opposite direction, Rätz et al. establish their design of a robotic hand by a set of clinical, anatomical, and mechanical requirements established before the development of the device. This novel clinical-driven robotic hand rehabilitation device is capable of fine haptic rendering, offering an effortless setup that supports physiological full flexion/extension of the fingers while providing high mechanical transparency. Lastly, Shi et al. develop a cable-driven three-degree-of-freedom wrist rehabilitation exoskeleton actuated by the distributed active semi-active (DASA) system. The proposed design has a larger workspace than current wrist rehabilitation training robots, able to cover a broader range of the activities of daily living, with an improved cable-driven design able to increase the effective torque and reduce the parasitic force.

### Control

Control strategies are quite significant to achieveimportant in achieving proper rehabilitation. In some cases, the rehabilitation routine imposes a pre-defined trajectory to be followed and, therefore, the control strategy must account for it. In others, the primary purpose is to regain neuroplasticity, and, thus, the patient's intention imposes control actions, and establishes how the system acts to achieve a proper trajectory. In this sense, Dalla Gasperina et al. present a cooperative control framework that promotes compliant motion and implements a variety of high-level rehabilitation modalities, including six actuation modes: passive, corrective, weight counterbalance, resistive, transparent, and hypergravity. The purpose is to change the haptic behavior perceived by a human when interacting with the rehabilitation robot by tuning different impedance control parameters. That variety of physical human-robot interactions helps the user to accomplish the task while exploiting physiological muscular activation patterns. Moving on to the work presented by Copaci et al., a new classifier for sEMG signals is presented. The algorithm is based on a Bayesian Neural Network in parallel with an Artificial Neural Network, which the results of which are connected in series with a Layer Recurrent Network. By doing so, the accuracy of the hand gesture recognition based on sEMG signals is improved. The authors' main purpose is to prove that the device control algorithm fits the patient's features and needs. The authors demonstrate that the proposed classifier could achieve high accuracy in hand gesture recognition. Last in this section, Castro et al. present a new approach for a 3D- printed hand prosthesis commanded by a simple sEMG system aided by a fully embedded computer vision system. The results show high percentages of accuracy, sensitivity, and specificity for grasping objects from neutral and pronated palmar grasp, tripod pinch, key grasp, and index finger extension gesture. This study shows that using a vision system is a promising alternative to traditional methods, as the pattern of grasping and manipulating objects is better defined.

### Validation

The assessment or validation of the designed prototype is a valuable step in developing rehabilitation devices before clinical testing. It allows the implementation of possible improvements before evaluating their actual effect on the target population. In this sense, Godfrey et al. present the first poly-articulated, electrically-actuated, and body-controlled artificial hand, called SoftHand Pro-Hybrid,; and compared its performance against the Hand Pro-Hybrid benchmark. Their results on a limited number of subjects to assess prototype's functionality, with and without limb loss, confirm the possibility of using hybrid solutions as a valid alternative to myoelectric control, especially in situations requiring high versatility of the device. Finally, the work by Sierra et al. present the assessment of the AGoRA Smart Walker in daily living scenarios with older adults with Parkinson's disease. This kind of device represents a valuable tool for assisting people with gait motor deficits. The actuators of the AGoRA Smart Walker adapt their assistance level to the subject's demands, improving the rehabilitation process. The authors also compared the performance of using their device by between two groups of older adults with different physical and cognitive characteristics (Parkinson's disease vs. other conditions).

### Clinical testing

A final step in assessing rehabilitation devices is their evaluation in a clinical setting. In this way, it is possible to evaluate the effects of the assistive device on a suitable target population. In this Research Topic, Chen et al. present a prospective, multi-center, and cross-over trial to evaluate the AIi-robot's safety, walking efficiency, donning and doffing time cost, and user satisfaction. They conclude that subjects with paraplegia below T6 level could ambulate safely and efficiently with that device, although its use should be learned under the guidance of experienced medical personnel. Marín-Méndez et al. present data from a two-arm, single-blinded, randomized, and controlled clinical trial. The objective was to evaluate the efficiency of a therapeutic massage robot (ADAMO) in reducing non-specific low back pain. Their main finding is that the ADAMO robot is at least as efficient as a regular treatment in reducing low back pain. However, it may be more beneficial for specific patients, such as those with who are overweight. Lastly, Koyama et al. present a prospective study for the Wearable Power-Assist Locomotor (WPAL). This device has been updated seven times, from the first validated prototype in 2005 to the latest in 2020. This study includes updated results from previous reports from July 2007 to December 2020 for 1785 different subjects. These results confirm that the WPAL improves walking independence for a wide range of spinal cord injury levels, and that further refinement of the WPAL will enable its long-term use at home.

### Adaptation strategies for future research in gait neuroprosthetics

To close this Research Topic, Koelewijn et al. expose an overview of research directions regarding interfaces with the peripheral and central nervous systems, and the requirements of interface- computing architectures. Their work guides the research on modular and adaptable interfaces that can assist as needed and process all data recorded in real-time while accounting for signal variations among subjects. Furthermore, biomechanical models and simulation techniques are pointed out to predict motion and interactions between the human and the rehabilitation device. This work summarizes the main challenges in designing and using neuroprosthetic devices.

## Conclusions and future perspective

The design of rehabilitation devices results from tight cooperation between engineers, industrial designers, physical therapists, physiatrists, and patients. Therefore, a lot of effort is required to achieve transparent and clinically efficient neuroprostheses and rehabilitation robots that assist neurological patients as needed. From the publications in this Research Topic, we foresee that biomechanical modeling and simulation will be increasingly used to optimize the design of such devices and study human-device interaction, as it allows virtual testing in different scenarios. Moreover, the design of efficient control strategies, and the development of data acquisition and processing techniques will lead to adequate and timed actuation to improve rehabilitation routines. The reduction in the dimensions and energy requirements of the actuation units will also lead to portable devices and increase the rehabilitation process at home. Lastly, the information gathered from biosignals or the possibility to interact with the neuromusculoskeletal system, such as in functional electrical stimulation or spinal cord stimulation, will make possible a new generation of rehabilitation devices able to overcome the current challenges faced in the rehabilitation of subjects with motor disabilities.

## Author contributions

FR-S wrote the first draft of the manuscript. All authors have made substantial and iterative contribution to the work and approved its final version for publication.

## Funding

The authors, in order of appearance, wish to thank the following support given by: the Ministry of Science and Innovation—Spanish Agency of Research (MCIN/AEI/10.13039/501100011033) through project PID2019-107491RB-I00 (FR-S), the National Council for Scientific and Technological Development (CNPq) though the Productivity Grant No. 311715/2021-4 (LL), the Ministry of Science and Innovation—Spanish Agency of Research (MCIN/AEI/10.13039/501100011033) and ERDF A way of making Europe through project RTI2018-097290-B-C33 (JF-L), and the European Union's Horizon 2020 Research and Innovation Programme as part of the European Research Council (ERC) Starting Grant INTERACT (803035) (MS).

## Conflict of interest

The authors declare that the research was conducted in the absence of any commercial or financial relationships that could be construed as a potential conflict of interest.

## Publisher's note

All claims expressed in this article are solely those of the authors and do not necessarily represent those of their affiliated organizations, or those of the publisher, the editors and the reviewers. Any product that may be evaluated in this article, or claim that may be made by its manufacturer, is not guaranteed or endorsed by the publisher.

